# The Editors’ Personal Biography of Professor Robert Anderson

**DOI:** 10.3390/jcdd8010006

**Published:** 2021-01-19

**Authors:** Nigel A. Brown, Deborah J. Henderson

**Affiliations:** 1 Molecular and Clinical Sciences Research Institute, St. George’s, University of London, London SW17 0RE, UK; 2 Bioscience Institute, Centre for Life, Newcastle University, Newcastle-upon-Tyne NE1 3BZ, UK; deborah.henderson@newcastle.ac.uk

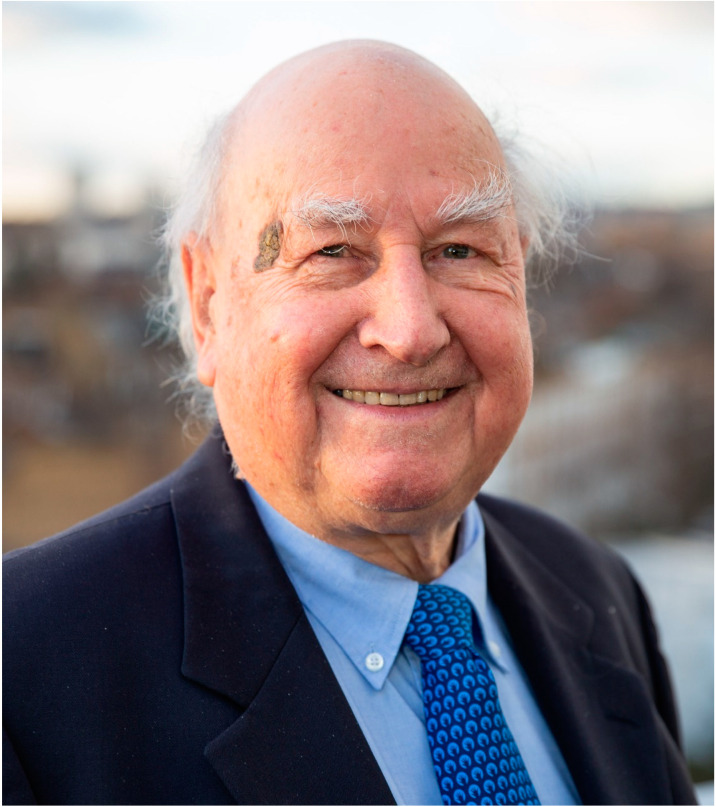



Robert (Bob) Henry Anderson was born in Wellington, Shropshire, UK, in 1942 and he completed his medical training in Manchester (UK) in 1966. In that same year, he married Christine, without whose constant support Bob would surely not have become the legendary figure he is today. Within 5 or 6 years, Bob had already published research in many of the areas that would define his career (Christine also had two children), and by 1979, he was rapidly and deservedly Professor of Paediatric Cardiac Morphology at the UK National Heart and Lung Institute in London.

Those first publications covered cardiac nerves (*Experientia*, 1971) [1]; the conduction system (*J. Anat.*, 1971) [2]; embryonic heart development (*Br. Heart J*., 1972) [3]; situs inversus (*Circulation*, 1972) [4] and congenital heart malformation (*Br. Heart J.*, 1972) [5]. One feature of these papers, which has continued ever since, is Bob’s acknowledgement of prescient contributions from the early researchers (and, of course, to correct them whenever necessary). In his first publication on the development of the heart, in 1972 [3], he cited papers from 1893, 1906, 1910, 1912 and 1914. He also started as he meant to continue, by naturally citing five of his own papers. Bob has never been afraid to commit his current understanding to paper and is always very willing to change that, in another publication, when new evidence arrives.

Over the following 50 years, Bob published more than 1000 peer-reviewed papers, making significant advances in these and in many other areas, establishing his reputation as a world-leading researcher. His seminal studies are cited by many of the contributions in this Special Issue—just a tiny reflection of Bob’s enduring influence on heart research. This would be sufficient for a major career, but there is much more to Bob’s contributions than just his substantial research record. He worked tirelessly to ensure a consistent and logical terminology for heart structures and their congenital abnormalities and to make this congruent with their developmental origins. Bob knows the correct identity and name of everything in and connected with the heart. Woe betide anyone who strays from this correctness, in print or in presentations.

In many ways, the most impactful aspect of Bob’s legacy is his education of and positive influence on several generations of clinicians and scientists, all across the wide field of heart research and practice. Again, many of those protégées appear as authors within this Special Issue and, indeed, functioned as Reviewers and Editors. From the beginning, in 1967, as an anatomy lecturer to medical students, Bob has been an enthusiastic and inspiring educator. Bob’s passion for the topic and his unfailing support for the learner are given freely, regardless of level, whether to the undergraduate in their first experiences, or to the expert in one of Bob’s masterclasses. Bob has toured the world throughout his career, lecturing in his inimitable pulpit style, and he continues to do so with regular visits to the USA (Chicago, Cincinnati, Denver, Milwaukee and Pittsburgh), Canada and India. Bob’s huge and infectious enthusiasm for the subject, and indeed, for life, is one aspect of his ability to make dear (in some cases, life-long) friends of his colleagues all over the world.

Those continuing regular visits illustrate Bob’s impressive longevity, not only in education but also research. Bob nominally retired in 2007, but it was difficult for many of us to discern any change. He now has Professorial and/or Consultant appointments in London, Newcastle, Manchester, Birmingham and Southampton in the UK, and in Charleston and Houston in the USA. Bob remains, as has always been the case, a prolific research collaborator. Over the past few decades of cellular and molecular technical advances, numerous developmental labs have sought out Bob as the go-to person to help them interpret their studies in the context of the intricate morphogenesis of the heart. He has a remarkable 4D model of embryonic heart anatomy in his brain, and these collaborations continue to be highly productive. Just as in his educational activities, Bob freely shares everything he knows in the research lab (which is worth bearing in mind when discussing your own work with Bob).

Bob is one of those enviable people who is good at everything they do, and he also has a wide-ranging store of deep knowledge. For example, he was a talented sportsman, so an impressive awareness of current UK sports (especially Manchester City) is not a surprise, but his encyclopaedic knowledge of US baseball is unexpected. Of course, Bob still plays a very creditable game of golf, on at least a weekly basis. Bob is also an accomplished pianist, a more-than-capable member of a classical duo or trio. Perhaps his greatest love is wine, for which he has impeccable taste and understanding. Bob has a very fine collection at home, and warehoused, and he is always generous in sharing this with the very many colleagues he and Christine have entertained and accommodated at their home in London.

During these more than 50 years of service to clinical practice, research and education, Bob has been honoured in many ways. Suffice to mention here just two recent awards: in 2017, he was elected as an honorary fellow of the European Congenital Heart Surgeon’s Association, and in 2019, he was accorded honorary membership of the Congenital Heart Surgeon’s Society of North America. We hope that this Special Issue is a fitting addition to these honours and that he knows that this reflects the high regard and affection that all of us involved in this collection have for the unique person that is Bob Anderson.
